# Monocrotophos Pesticide Decreases the Plasma Levels of Total 3,3′,5-Triiodo-l-Thyronine and Alters the Expression of Genes Associated with the Thyroidal Axis in Female Goldfish (*Carassius auratus*)

**DOI:** 10.1371/journal.pone.0108972

**Published:** 2014-09-30

**Authors:** Xiaona Zhang, Hua Tian, Wei Wang, Shaoguo Ru

**Affiliations:** College of Marine Life Sciences, Ocean University of China, Qingdao, China; Ecole Normale Supérieure de Lyon, France

## Abstract

Our recent study showed that monocrotophos (MCP) pesticide disrupted the hypothalamic-pituitary-thyroid (HPT) axis in male goldfish (*Carassius auratus*); however, the effects of MCP on the thyroid system in female goldfish are remain unclear. In the present study, plasma thyroid hormone (TH) and thyroid-stimulating hormone (TSH) levels were evaluated in female goldfish exposed to 0.01, 0.10, and 1.00 mg/L of 40% MCP-based pesticide for 21 days in a semi-static exposure system. Expression profiles of HPT axis-responsive genes, including transthyretin (*ttr*), deiodinases (*d1*, *d2*, and *d3*), *tshβ*, thyrotropin-releasing hormone (*trh*), and corticotrophin-releasing hormone (*crh*), were determined. The results indicated that MCP decreased the plasma levels of total 3,3′,5-triiodo-l-thyronine (TT_3_) and the ratio of TT_3_ to total 3,3′,5,5′-l-thyroxine (TT_4_), and induced alternative expression of TH-related genes. Exposure to 0.01 and 0.10 mg/L MCP pesticide resulted in the up-regulation of *ttr* mRNA. The reduction of plasma TT_3_ levels was partly attributed to an increase in the metabolism of T_3_ in the liver, as revealed by the highly elevated hepatic *d1* and *d3* mRNA levels in the MCP treatment groups, and the expression of hepatic *d3* showed a negative correlation with the plasma TT_3_/TT_4_ levels in females. Moreover, the plasma TSH levels were lower in females exposed to 0.01 and 0.10 mg/L MCP pesticide, whereas the up-regulation of *tshβ* mRNA levels was compensated by the decreased plasma TT_3_ levels. These results indicated that MCP had the potential to influence several pathways of HPT axis homeostasis in female goldfish.

## Introduction

In fish, growth and reproduction are, at least partly, under the control of thyroid hormones (THs), 3,3′,5,5′-l-thyroxine (T_4_) and 3,3′,5-triiodo-l-thyronine (T_3_) [1], [2]. Considering that reproduction in females from the early development of follicles to oocyte maturation and ovulation involves a significant investment of energy to support the related physiological functions, the reproductive-related allocation of energy is particularly important during ovarian development in oviparous species, including fish. Since THs have profound effects on energy metabolism, for example, they are the major regulators of oxidative energy metabolism at the level of the mitochondria in teleost fish [3], they should invariably be involved in the multifactorial regulation of metabolism associated with reproduction. They might also act as direct modulators of the reproductive cycle. A previous study on goldfish (*Carassius auratus*) has shown that T_4_ could act synergistically with gonadotropin to influence ovarian development by increasing ovarian sensitivity to gonadotropic stimulation [4]. Moreover, the expression of steroidogenic enzymes and steroid receptors in goldfish could be modulated by THs [5].

Monocrotophos (MCP; CAS number, 6923-22-4), an organophosphorus pesticide, has been banned in developed countries due to its high toxicity; however, it is still used extensively in agricultural practices in developing countries such as Pakistan, China, and India, where high pesticide residue levels have led to excessive MCP levels in the environment. For example, the concentrations of MCP detected in water sources in China and rain water in India were 0.165 and 4 µg/L, respectively [6], [7]. Anjum and Malik [8] reported the presence of organophosphorus pesticide in the industrial wastewater around Lucknow, India, and the concentration of MCP was determined to be 8.32±3.9 ng/mL. Our recent study showed that the MCP pesticide exhibited thyroid-disrupting effects by interfering with the thyroidal axis, resulting in decreased plasma total T_3_ (TT_3_) levels in male goldfish [9]. Further, a gender difference in the thyroid system in response to metolachlor has been reported by Jin et al. [10], and the authors considered that endogenous sex hormones might modify the response of the thyroid system to the environmental chemical. In our previous study, MCP was found to interfere with the reproductive axis *via* several pathways, thereby inducing increases in plasma 17*β*-estradiol (E_2_) levels and decreases in testosterone (T) levels in both male and female goldfish [11], [12]. Balanced plasma TH levels are known to be crucial for normal reproductive function; in mammals, both hyperthyroidism and hypothyroidism were found to result in reproductive impairment and lower fertility [13]. Considering gender differences in the response of the thyroid endocrine system in fish exposed to environmental pollutants, the thyroid disruption effects of MCP in females might be speculated to be similar to those found in males. The effects of MCP on the thyroid endocrine system in females, however, remain unclear.

THs are synthesized and secreted by the thyroid follicles under the control of the HPT axis. In teleosts, the thyrotropin-releasing hormone (TRH) and/or corticotrophin-releasing hormone (CRH), released from the hypothalamus, coordinate the HPT axis function by controlling the release of thyrotropin (TSH) from the pituitary, which could stimulate TH synthesis and release [1], [14]. Most of the plasma THs in fish are bound to transthyretin (TTR), a specific TH transport protein in teleosts [15], and only free hormones can enter target cells to elicit a response. In the liver and some other peripheral tissues, three types of deiodinases (type I, D1; type II, D2; and type III, D3) are known to control the conversion of T_4_ to the more physiologically active T_3_ or the production of metabolically inactive counterparts [16]. Such complex regulatory pathways are involved in thyroid homeostasis. Therefore, environmental chemicals can act at multiple stages in the HPT axis.

In the present study, following administration of 40% MCP pesticide to female goldfish, plasma TH levels, including TT_3_, total T_4_ (TT_4_), and the physiologically relevant free T_3_ (FT_3_) and free T_4_ (FT_4_) levels, were determined, and the changes in endocrine-mediated responses along the HPT axis were evaluated, including the regulation of TRH and CRH, synthesis and secretion of TSH, and expression of deiodinases (*d1*, *d2*, and *d3*) and transthyretin (*ttr*) genes.

## Materials and Methods

### Pesticide

MCP pesticide (3-hydroxyl-*N*-methyl-*cis*-crotonamidedimethyl phosphate, 40% water-soluble preparation) was purchased from the Qingdao pesticide factory in China. The concentration on the label was 40%, which was consistent with the actual concentration determined by gas chromatography (40±0.1%) [17]. The half-life of MCP is approximately 66 days at pH 7.0 and 20°C [18].

### Fish exposure and sample protocols

Sexually mature goldfish (*C. auratus*) with mature and fully developed gonads (8.7±0.8 cm standard length; 21.2±4.1 g wet weight; 6.8±0.3% gonadal somatic index) were obtained from a local dealer in Qingdao, PR China, and sampled in late spring. Fish were handled according to the National Institute of Health Guidelines for Handling and Care of Experimental Animals. The animal utilization protocol was approved by the Institutional Animal Care and Use Committee of the Ocean University of China. Fish were maintained in a 70-L aquarium containing 50-L dechlorinated tap water at ambient temperature (18±2°C) with dissolved oxygen content of 7.0±0.1 mg/L and were fed small shrimp daily.

After the female goldfish were acclimated to laboratory conditions for 2 weeks, they were exposed to nominal 0.01, 0.10, and 1.00 mg/L 40% MCP pesticide (4, 40, and 400 µg/L equivalent levels of pure MCP), the concentrations of which were 1/10,000; 1/1,000; and 1/100 of the 96-h LC_50_ [11], respectively. The experiments were conducted in the same 70-L aquarium containing 50-L dechlorinated tap water by using a semi-static toxicity test (20-L water renewal daily to constantly maintain the MCP concentration). Each group of fish was exposed in three aquaria (seven fish/tank) and a control (dechlorinated tap water) was included in the exposure design. Less than 10% mortality was observed in all the treatments during the experiment, and abnormal behaviors such as unusual swimming pattern or bumping against the tank were observed in the group exposed to the highest dose.

After 21 days of exposure, all fish were anesthetized with 75 mg/L MS-222 (Sigma, St. Louis, MO, USA) and sampled between 9∶00 and 11∶00 h to avoid the possible influences of diurnal fluctuations in hormone levels on the results [19]. Fish from each group were divided into two subgroups at the time of sampling. One subgroup of 9–10 fish was sampled to collect 0.6–0.8 mL blood for TH measurements, whereas the remaining fish were used to collect blood with the same volume for TSH and cortisol measurements. Blood was collected from the caudal vein by using chilled heparinized syringes and maintained on ice. Plasma samples were obtained after centrifugation at 1000×*g* for 10 min and stored at −80°C until the hormone assay could be performed. The liver, pituitary, and hypothalamus tissues were dissected (n = 9), frozen in liquid nitrogen, and then stored at −80°C for the quantification of gene expression by real-time polymerase chain reaction (PCR).

### Hormone assay

The plasma levels of TT_3_, TT_4_, FT_3_, FT_4_, TSH, and cortisol were measured using radioimmunoassay (RIA) by using commercially available kits following the protocols provided by the manufacturers (Beijing North Institute of Biological Technology, Beijing, China). The RIA kits for human TT_3_, TT_4_, FT_3_, FT_4_, TSH, and cortisol were validated for use with goldfish samples by showing parallelism between a series of diluted and spiked samples in relation to the standard curve included with the assay kits. Standards and samples were added to test tubes in duplicate. The assay detection limits were 0.05 ng/mL for T_3_, 2 ng/mL for T_4_, 0.06 pg/mL for FT_3_, 0.23 pg/mL for FT_4_, 0.1 µIU/mL for TSH, and 1 ng/mL for cortisol. The inter- and intra-assay coefficients of variation for all the above-mentioned hormones were <10% and <15%, respectively.

### Gene expression analysis

Total RNA from each tissue was isolated using the phenolic reagent TRIzol (Invitrogen, Carlsbad, CA, USA) according to the manufacturer’s protocol. The extracted RNA was measured by spectrometry at OD_260/280_ before treatment with DNase I (Promega, Madison, WI, USA). Equal amounts of RNA (1 µg) were reverse-transcribed into cDNA in 20-µL reactions containing 10 pmol oligo(dT)_20_, 4 µL 5× RT Buffer, 2 µL dNTP mixture, 10 U RNase inhibitor, and 1 µL ReverTra Ace (Toyobo, Tokyo, Japan). Reverse transcription reactions were conducted in a Bio-Rad DNA Thermal Cycler (Hercules, CA, USA) at 42°C for 20 min and terminated for 5 min at 85°C.

Oligonucleotide primers were designed for the specific amplification of *tshβ*, *trh*, *crh*, *ttr*, *d1*, *d2*, *d3*, *β*-actin, and 18S rRNA by using the Primer Premier 5.0 software (PREMIER Biosoft Int., Palo Alto, USA) and the sequences for goldfish available from the GenBank database (Table 1). The amplifications were performed using an Eppendorf MasterCycler ep *RealPlex* ^4^ (Eppendorf, Wesseling-Berzdorf, Germany). Parallel PCR analyses were conducted to amplify the cDNA of the target and reference genes. Real-time PCR was performed in 20-µL reaction mixtures containing 1× SYBR Premix Ex *Taq*, 0.4 µM of each primer, 0.4 µL of ROX reference Dye (Takara Bio Inc., Shiga, Japan), and 4 µL of first-strand cDNA (template). The thermal profile was 95°C for 30 s, followed by 40 cycles of 95°C for 5 s and 60°C for 30 s. Amplification of a single product was ensured by performing a melting curve analysis by using the PCR products obtained at the end of each PCR run. In addition, 2% agarose gel electrophoresis of the PCR products was performed to confirm the presence of single amplicons having the predicted sizes (data not shown). The *β*-actin and 18S rRNA transcripts were used as housekeeping genes to standardize the results and eliminate variations in mRNA and cDNA quantity and quality. Neither the *β*-actin nor 18S rRNA levels were affected by any of the experimental conditions used in the study. For each reaction, the relative target gene mRNA expression levels were normalized to the geometric mean of *β*-actin and 18S rRNA expression levels by using the formula 2^−ΔΔCt^ and plotted on a logarithmic scale [20].

**Table 1:** Nucleotide sequences of the primers used for real-time polymerase chain reaction and product sizes.

Gene	GenBank accession no.	Primer sequence (5′–3′)	Amplicon size (bp)
*tshβ*	AB003584	F: TGGCTGTCAACACCACCATCTG	94
		R: CCCCTTTGAACCAGGAAACGAG	
*trh*	AB179819	F: GAACAAGAGTCGCTGTAAGGGAG	102
		R: GGATGCTGTGGAAAACAATGAAC	
*crh*	AF098629	F: CCAGAATTATCTCCGATCCCCAG	109
		R: CCCAACTTTCCCCTCCAACAG	
*ttr*	EU313781	F: TTCAGATGCTCACTGCCCTTTG	103
		R: CCCACCTTGTTCTTGGCGATAG	
*d1*	EU313785	F: GGCAGATTTCCTTGTTGTTTACC	112
	EF190702	R: CGCAGCCAGTCTCTCCTCC	
*d2*	EU313786	F: CAAACTCCAAGGTGGTGAAGGT	86
	EF190703	R: GTCCAGCAGATGGCACTCGT	
*d3*	EF190704	F: GCTGTTCCTGGATTTCCTGTGC	163
	EU313787	R: GAAGAAGTCCAGCTTGTGTCCGT	
*β-*actin	AB039726	F: GAAACTGGAAAGGGAGGTAGC	115
		R: CTGTGAGGGCAGAGTGGTAGA	
18S rRNA	AF047349	F: AGAAACGGCTACCACATCCAAG	169
		R: GCACCAGATTTGCCCTCCAG	

Goldfish contain duplicate genes encoding for D1, D2, and D3, and the primer pairs amplify the two genes.

### Statistical analysis

The experimental data were presented as the means ± standard deviations, and the differences between the control and each exposure group were evaluated using one-way analysis of variance (ANOVA) followed by Tukey’s test. Before parametric analysis, assumptions of normality and homogeneity of variances were assessed using probability plots and normality tests. Pearson’s correlation coefficient was used to calculate the relationship between the expression of certain genes involved in the HPT axis and the plasma TH levels. Values were considered significant when 0.01<*P*<0.05 and highly significant when *P*<0.01.

## Results

### Effects of MCP pesticide on plasma TT_3_ and TT_4_ levels and the ratio of TT_3_ to TT_4_

**Figure 1:**
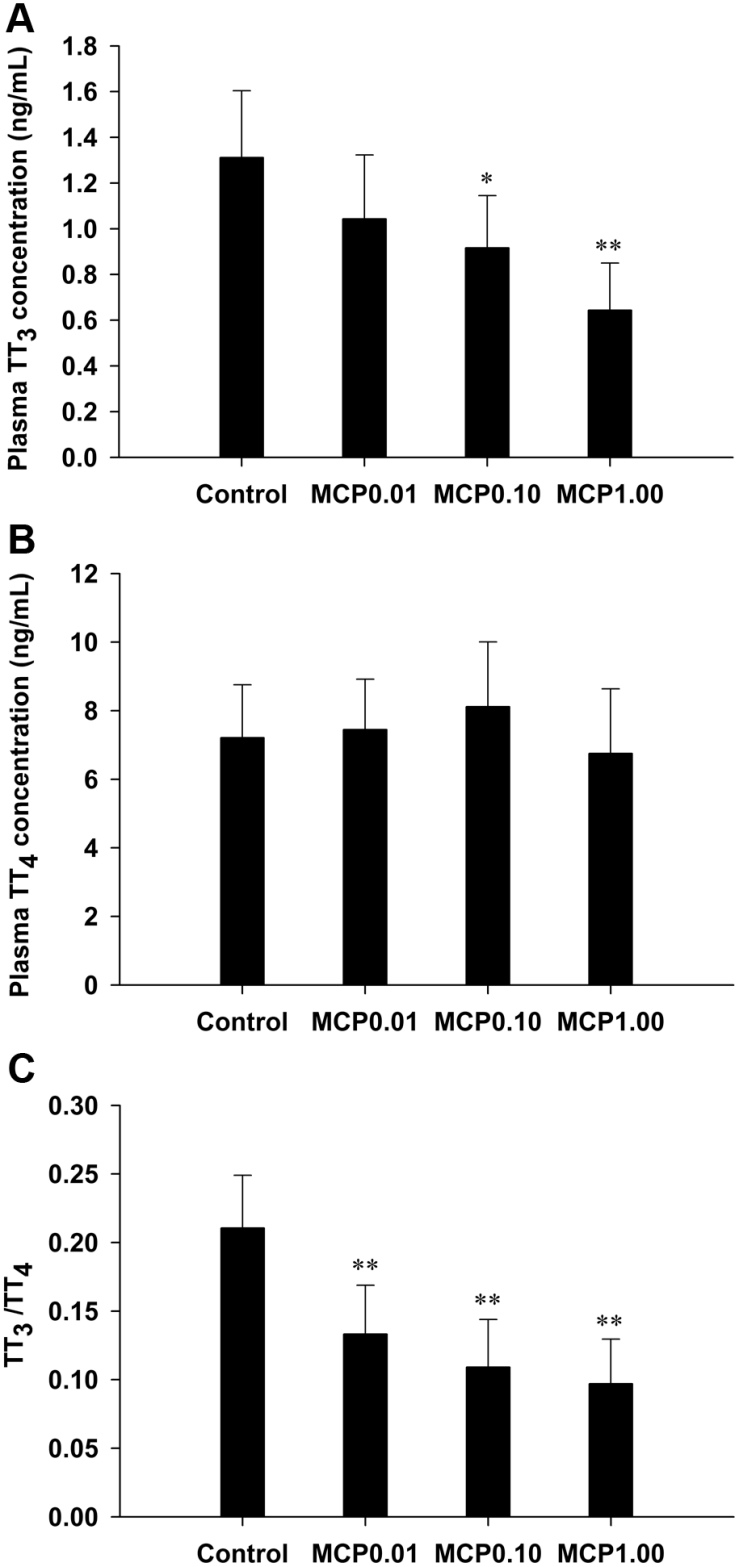
Quantification of plasma total 3,3′,5-triiodo-l-thyronine (T_3_) and total 3,3′,5,5′-l-thyroxine (T_4_) levels in female goldfish exposed to 0, 0.01, 0.10, and 1.00 mg/L 40% monocrotophos (MCP) pesticide. (designated C, MCP0.01, MCP0.10, and MCP1.00, respectively). The data are presented as the means ± standard deviations (n = 9). Asterisks indicate statistically significant differences from the control group (*0.01<*P*<0.05, **P<0.01).

The TT_3_ content in the plasma of the control female goldfish was 1.31±0.29 ng/mL, whereas that in the fish exposed to 0.10 and 1.00 mg/L MCP pesticide was significantly decreased by 30% to 0.92±0.23 ng/mL (0.01<*P*<0.05) and 51% to 0.64±0.21 ng/mL (*P*<0.01), respectively (Fig. 1A). At the end of the MCP treatment, the TT_4_ content was 7.21±1.55 ng/mL in the control group, and there was no significant change in the TT_4_ content of any of the MCP pesticide-treated groups (Fig. 1B). There was a dose-related decrease in the ratio of TT_3_ to TT_4_ after MCP treatment (*P*<0.01; Fig. 1C).

### Effects of MCP pesticide on plasma FT_3_ and FT_4_ levels and hepatic *ttr* mRNA expression

**Figure 2:**
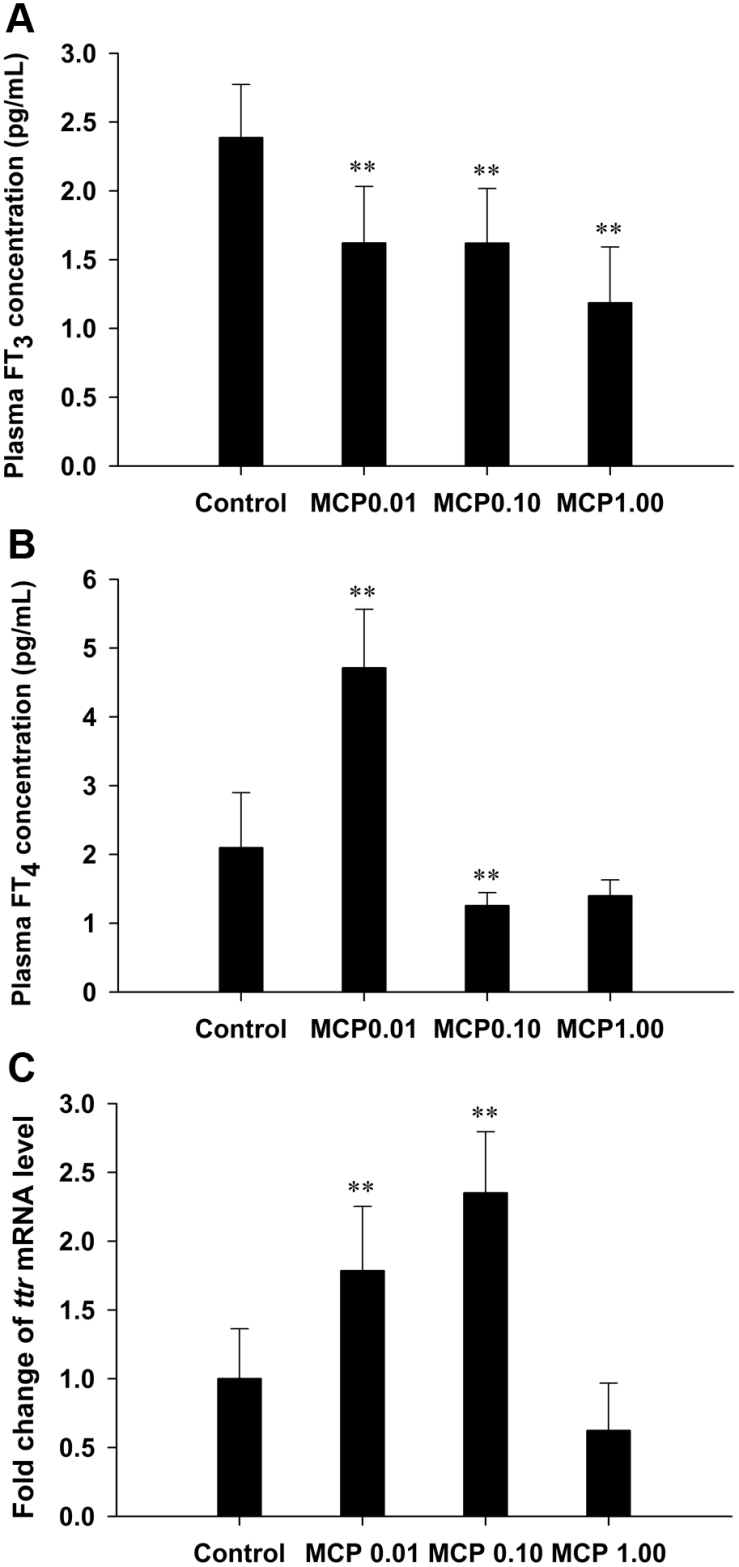
Quantification of plasma free 3,3′,5-triiodo-l-thyronine (T_3_) and free 3,3′,5,5′-l-thyroxine (T_4_) contents and the relative mRNA expression levels of hepatic transthyretin (*ttr*) in female goldfish exposed to 0, 0.01, 0.10, and 1.00 mg/L 40% monocrotophos (MCP) pesticide. (designated C, MCP0.01, MCP0.10, and MCP1.00, respectively). For panel C, fold change (Y axis) represents the expression of the target gene mRNA relative to that of the control group (equals 1 by definition). The data are presented as the means ± standard deviations (n = 9). Asterisks indicate statistically significant differences from the control group (*0.01<*P*<0.05, **P<0.01).

The basal FT_3_ levels in the plasma of the control female goldfish were 2.39±0.39 pg/mL; they were significantly reduced by 32%, 32%, and 50% in the 0.01, 0.10, and 1.00 mg/L MCP pesticide-treated groups, respectively (*P*<0.01; Fig. 2A). Plasma FT_4_ levels in the control females were 2.10±0.80 pg/mL; they were significantly increased to 4.71±0.85 pg/mL in the 0.01 mg/L MCP pesticide-treated group (*P*<0.01) but decreased to 1.26 pg/mL in the 1.00 mg/L MCP pesticide-treated group (*P*<0.01; Fig. 2B). Furthermore, the mRNA expression of hepatic *ttr* was significantly higher after treatment with 0.01 and 0.10 mg/L of MCP pesticide (*P*<0.01), whereas there was no significant difference in the hepatic *ttr* mRNA expression between the group treated with the highest dose and the control group (Fig. 2C).

### Effects of MCP pesticide on *d1*, *d2*, and *d3* mRNA expression levels in the liver, brain, and kidney

**Figure 3:**
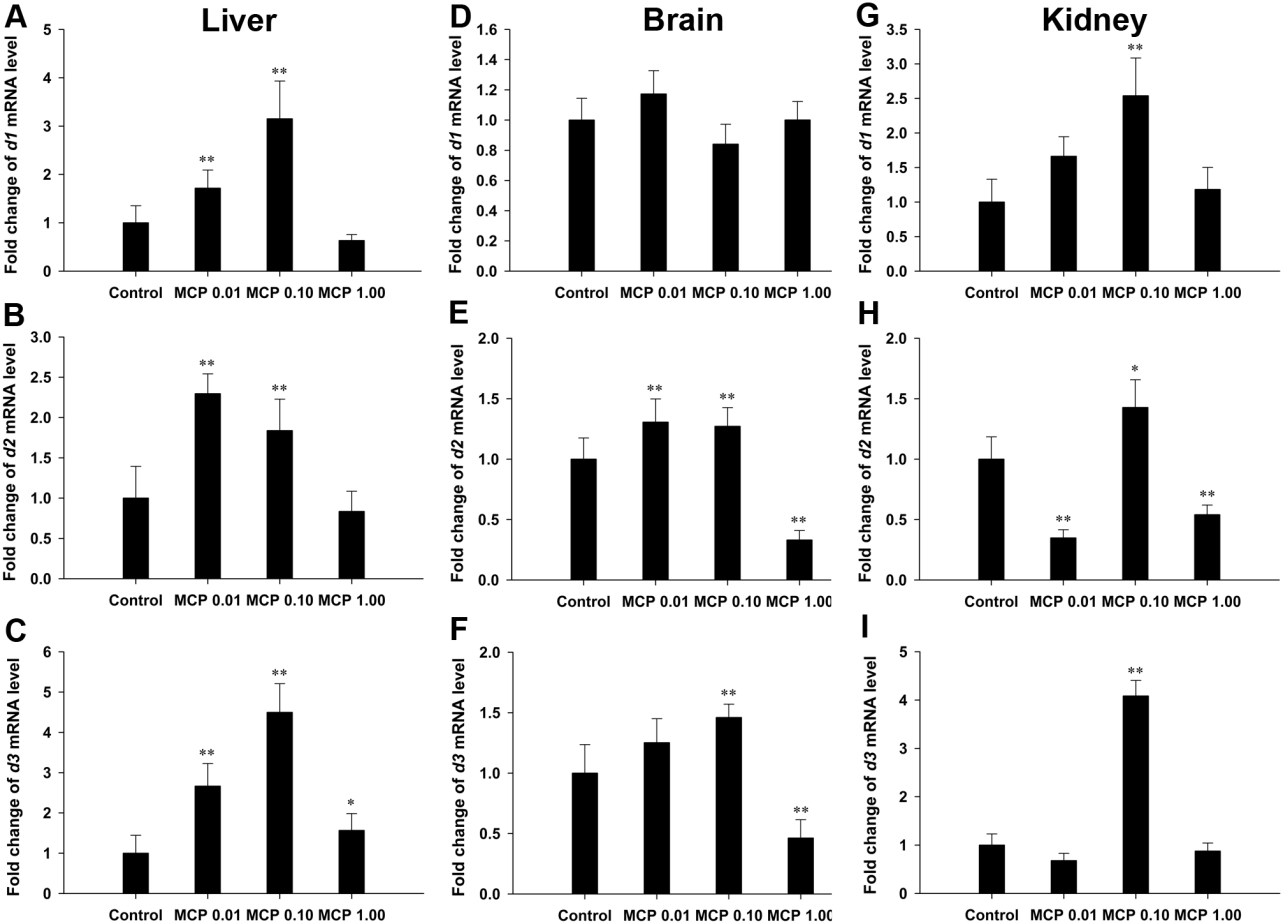
Relative mRNA expression levels of types I, II, and III deiodinases (*d1*, *d2*, and *d3*) in the liver, brain, and kidney of female goldfish exposed to 0, 0.01, 0.10, and 1.00 mg/L 40% monocrotophos (MCP) pesticide. (designated C, MCP0.01, MCP0.10, and MCP1.00, respectively). Fold change (Y axis) represents the expression of the target gene mRNA relative to that of the control group (equals 1 by definition). The data are presented as the means ± standard deviations (n = 9). Asterisks indicate statistically significant differences from the control group (*0.01<*P*<0.05, **P<0.01).

The *d1* mRNA levels in the liver were significantly increased by 0.72- and 2.15-fold and the hepatic *d2* mRNA expression was significantly up-regulated by 1.30- and 0.84-fold after treatment with 0.01 and 0.10 mg/L MCP pesticide, respectively (*P*<0.01), relative to those in the control (Fig. 3A and 3B). Hepatic *d3* mRNA levels were significantly higher in all the MCP-treated groups, especially in the 0.01 and 0.10 mg/L MCP pesticide-treated groups (2.66- and 4.50-fold higher, respectively), than those in the control (*P*<0.01; Fig. 3C).

With regard to the gene transcription level of deiodinase in the brain (Fig. 3D–3F), there was no significant difference in the *d1* gene transcription in any of the MCP-exposed groups. MCP pesticide exposure significantly stimulated *d2* gene transcription in the 0.01 mg/L and 0.10 mg/L groups (*P*<0.01) and *d3* gene transcription in the 0.10 mg/L group (*P*<0.01). However, the transcription of both *d2* and *d3* genes was significantly inhibited after exposure to 1.00 mg/L MCP pesticide (*P*<0.01).

Transcription of the *d1* gene in the kidney was significantly up-regulated after exposure to 0.10 mg/L MCP (*P*<0.01; Fig. 3G). The transcription of *d2* was significantly down-regulated after exposure to 0.01 and 1.00 mg/L MCP pesticide, but was stimulated in the 0.10 mg/L group (0.01<*P*<0.05; Fig. 3H). The expression levels of *d3* mRNA in the kidney were significantly up-regulated only in the 0.10 mg/L MCP-exposed group (4.0-fold, *P*<0.01; Fig. 3I).

### Effects of MCP pesticide on pituitary *tshβ* mRNA expression and plasma TSH levels

**Figure 4:**
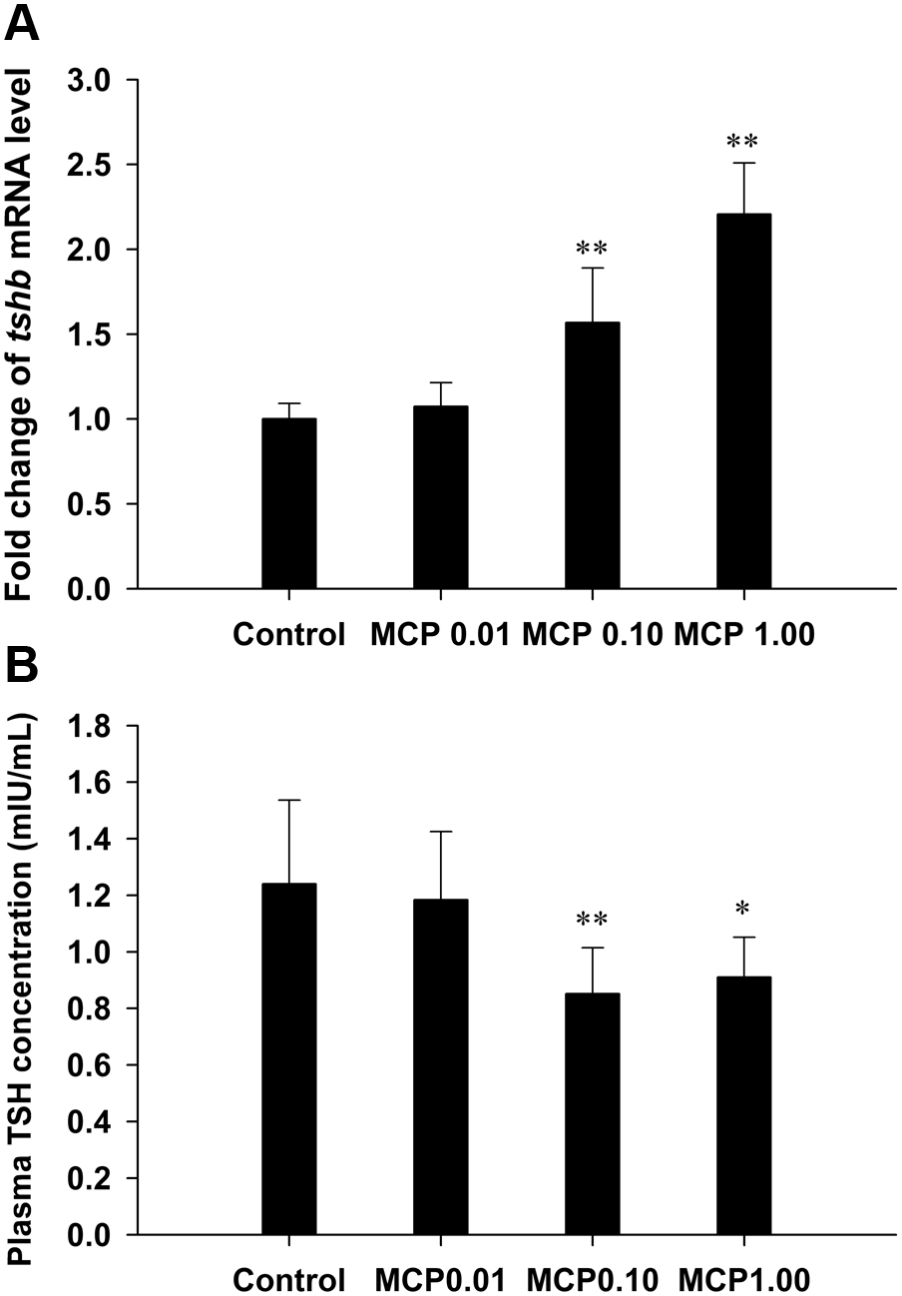
Relative mRNA expression levels of pituitary thyroid-stimulating hormone *β* subunit (*tshβ*) and quantification of plasma TSH content in female goldfish exposed to 0, 0.01, 0.10, and 1.00 mg/L 40% monocrotophos (MCP) pesticide. (designated C, MCP0.01, MCP0.10, and MCP1.00, respectively). For panel A, fold change (Y axis) represents the expression of the target gene mRNA relative to that of the control group (equals 1 by definition). The data are presented as the means ± standard deviations (n = 9). Asterisks indicate statistically significant differences from the control group (*0.01<*P*<0.05, **P<0.01).

The mRNA expression of pituitary *tshβ* was significantly up-regulated in both the 0.10 and 1.00 mg/L MCP pesticide-treated groups (*P*<0.01; Fig. 4A). In contrast, the plasma TSH levels in the 0.10 and 1.00 mg/L MCP pesticide-treated groups were markedly decreased to 0.85±0.16 µIU/mL (*P*<0.01) and 0.91±0.14 µIU/mL (0.01<*P*<0.05), respectively, compared to those in the control group (1.24±0.30 µIU/mL).

### Effects of MCP pesticide on hypothalamic *trh* and *crh* mRNA and plasma cortisol levels

**Figure 5:**
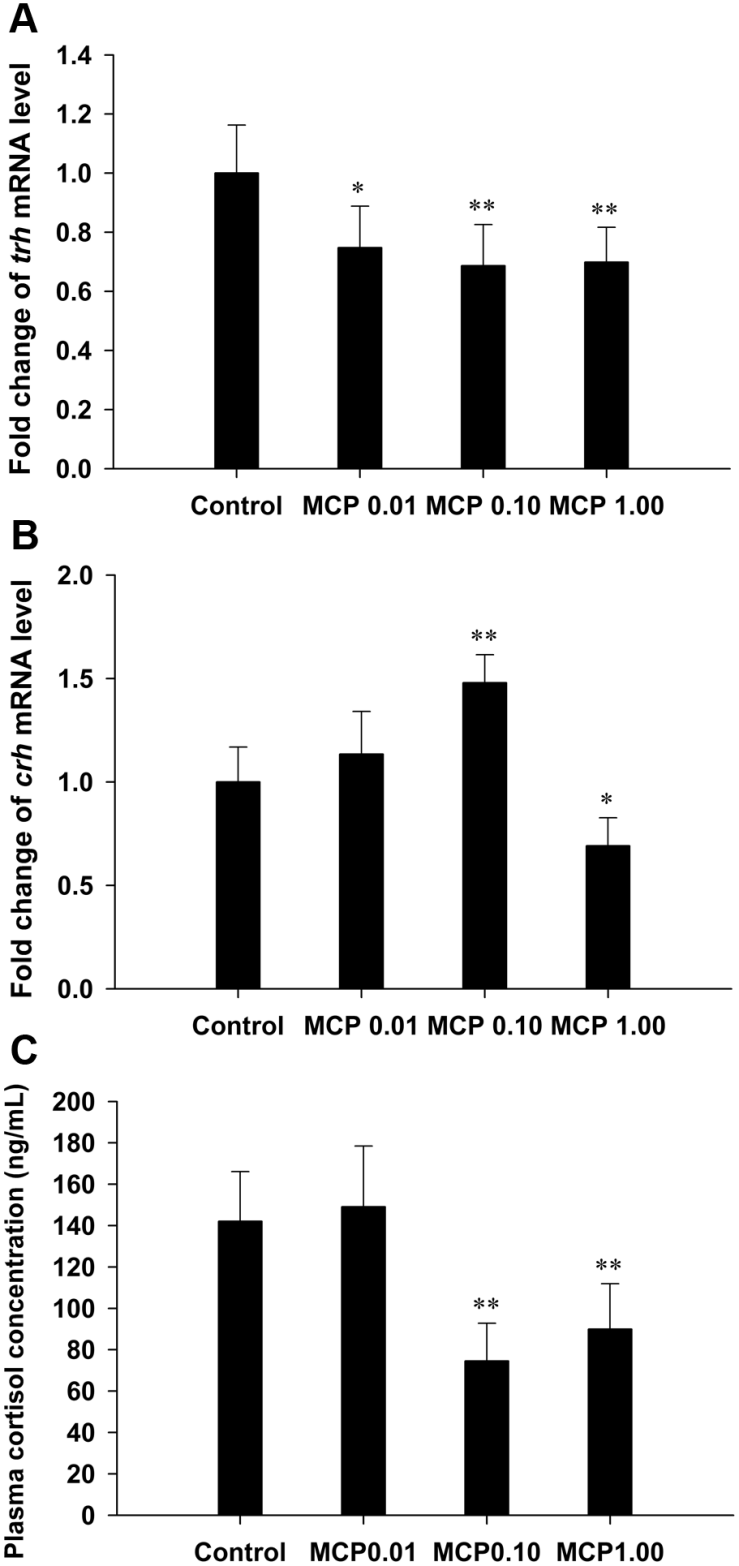
Relative mRNA expression levels of thyrotropin-releasing hormone (*trh*) and corticotrophin-releasing hormone (*crh*) in the hypothalamus glands and quantification of plasma cortisol content in female goldfish exposed to 0, 0.01, 0.10, and 1.00 mg/L 40% monocrotophos (MCP) pesticide. (designated C, MCP0.01, MCP0.10, and MCP1.00, respectively). For panels A and B, fold change (Y axis) represents the expression of the target gene mRNA relative to that of the control group (equals 1 by definition). The data are presented as the means ± standard deviations (n = 9). Asterisks indicate statistically significant differences from the control group (*0.01<*P*<0.05, **P<0.01).

The expression of hypothalamic *trh* was significantly down-regulated after treatment with the MCP pesticide (Fig. 5A). The transcription of the *crh* gene was significantly stimulated after treatment with 0.10 mg/L MCP pesticide (*P*<0.01), but inhibited in the 1.00 mg/L MCP pesticide-treated group (0.01<*P*<0.05; Fig. 5B). The measured plasma cortisol content was 142.01±24.11 ng/mL in the control female goldfish and was significantly decreased to 74.53±18.26 ng/mL and 89.88±22.05 ng/mL in the 0.10 and 1.00 mg/L MCP pesticide-treated groups, respectively (*P*<0.01; Fig. 5C).

### The correlation coefficient for plasma THs levels and the expression of certain genes related to the HPT axis

The correlation coefficient for plasma THs levels and the expression of certain genes associated with the HPT axis are shown in Table 2. Significant correlations of the ratio of TT_3_ to TT_4_ with hepatic *d3* expression, TT_3_ with pituitary *tshβ* expression, and FT_4_ with hepatic *ttr* expression were found.

**Table 2:** Pearson correlation coefficients for the plasma thyroid hormone levels and the expression of certain genes related to the hypothalamus-pituitary-thyroid axis.

Paired samples	Correlation coefficient
TT_3_/TT_4_ vs. hepatic *d1*	−.189
TT_3_/TT_4_ vs. hepatic *d2*	−.232
TT_3_/TT_4_ vs. hepatic *d3*	−.384*
TT_3_ vs. pituitary *tshβ*	−.469*
TT_3_ vs. hypothalamus *trh*	.356
TT_3_ vs. hypothalamus *crh*	.025
FT_3_ vs. hepatic *ttr*	−.069
FT_4_ vs. hepatic *ttr*	.396*

*Statistically significant at 0.01<*P*<0.05.

## Discussion

An increasing number of studies have reported that groups of pesticides, including acetochlor, amitrole, and metolachlor, have the potential to influence several steps in HPT axis homeostasis and to induce THs disturbance in adult fish, particularly with respect to sex differences occurring in response to chemical-induced thyroid system disruption [10], [21], [22]. For example, Li et al. [21] showed that TH-related genes such as malic enzyme and sodium iodide symporter were significantly down-regulated in the brains of the rare minnow *Gobiocypris rarus*, and that the expression of these genes in females was more sensitive to acetochlor than that in males. Recently, we found that a 21-d exposure to MCP pesticide caused significant decreases in plasma TT_3_ levels and TT_3_-to-TT_4_ ratios in male goldfish [9]; however, whether similar effects occur in females is not clear.

In the present study, although the plasma levels of TT_4_ remained unchanged, those of TT_3_, FT_4_, and FT_3_ in female goldfish were significantly altered after a 21-day exposure to MCP pesticide, suggesting a failed adaptation and auto-regulation of THs homeostasis. Almost all THs circulating in the plasma are bound to transporter proteins, and the equilibrium of TH binding to the plasma proteins determines the concentration of free THs within the plasma [23]. TTR, which is primarily a secretory product of the liver, has been proposed to be the major TH-carrier protein that binds THs and transports them to target tissues in fish [24]–[26]. In our study, TTR gene expression was up-regulated after exposure to 0.01 and 0.10 mg/L MCP pesticide. This up-regulation might have resulted in higher TTR mRNAs and thus TTR proteins, leading to decreases in plasma FT_3_ and/or FT_4_ levels. Moreover, the expression of *ttr* showed a positive correlation with plasma FT_4_ levels in females. Notably, less than 1% of plasma TT_4_ is free with 99% reversibly bound to plasma proteins [23]. Indeed, changes of such small amount of FT_4_ contents may not represent a dynamic circulating TT_4_ reservoir and vice verse. For example, in brown trout (*Salmo trutta*) fed diets enriched with *β*–Tetrabromoethylcyclohexane for 56 days, there was no significant difference among treatments in FT_4_, but TT_4_ was significantly reduced in the high dose group relative to the control [27]. In the 0.01 mg/L MCP group in particular, one possible explanation for the increased plasma FT_4_ levels could be that feedback systems attempt to respond to the reduction in plasma TT_3_ levels. However, the stimulatory effects of 0.01 mg/L MCP pesticide on plasma FT_4_ levels in females were not observed in males [9], indicating that the thyroidal system in female goldfish is more sensitive to MCP than that of males. In the group treated with the highest dose of MCP pesticide, the total TH level was more readily responsive to the changes in free hormone levels, since TTR gene expression was not stimulated in this group.

Among the thyroid follicle secretions, T_4_ is the predominant circulating hormone in the blood of fish, and T_3_ appears to be produced largely by enzymatic deiodination of T_4_ in the peripheral tissues [28]. Consequently, plasma T_3_ levels mostly decline due to a drop in thyroidal T_4_ production and secretion and/or changes in the peripheral TH metabolism [29]. Our finding that decreases in plasma TT_3_ levels along with relatively stable plasma TT_4_ levels suggested possible changes in peripheral TH deiodination or metabolism. Iodothyronine deiodinases play a crucial role in the mechanism of TH biotransformation in extra-thyroidal tissues. Conversion from T_4_ to T_3_ is mediated by outer-ring deiodination (ORD). Both inner-ring deiodination (IRD) and ORD are involved in the inactivation of T_4_ to 3,3′,5′-triiodo-l-thyronine (rT_3_) and T_3_ and rT_3_ to 3,3′-diiodo-l-thyronine (3,3′-T_2_) [16], [30]. Three types of deiodinases have been identified in teleosts: type I (D1) has both ORD and IRD activities, whereas types II (D2) and III (D3) have ORD and IRD activity, respectively [31]–[34]. At the end of the 21-day exposure to 0.01 and 0.10 mg/L MCP pesticide, the transcription of all three types of deiodinases was stimulated in the liver: changes in the expression of *d1* and *d3* mRNA were higher than those of *d2* mRNA. Further, in the 1.00 mg/L treatment, only hepatic *d3* mRNA levels were significantly up-regulated. Van der Geyten et al. [35] showed that the changes in hepatic deiodinases activities tend to be consistent with those of their mRNA levels, indicating pre-translated regulation of the hepatic deiodinases. Both D1 and D3 are involved in the metabolism of THs. D3 catalyzes the degradation of T_4_ and T_3_ to inactive metabolites and could protect tissues from an excess of biologically active TH, namely, T_3_ [36]. Although D1 also has the ability to degrade T_3_, it is less effective than D3, and its preferred substrate is rT_3_ [37]. Therefore, the stimulated enzymatic metabolism of T_3_ in the liver could cause a reduction in the levels of circulating TT_3_. Consistently, a significant negative correlation between hepatic *d3* mRNA expression and the ratio of TT_3_ to TT_4_ was observed. It is also worth noting that, in the highest dose group, alterations of gene expression of the three types of deiodinases in females were not consistent with those in males in which hepatic *d1* and *d2* mRNA levels were significantly down-regulated, whereas *d3* mRNA levels were not affected [9].

Although liver is considered to be the main peripheral source of circulating T_3_, TH deiodination also occurs in other extra-thyroidal tissues such as brain and kidney; however, the available T_3_ derived from these tissues is primarily utilized by the same tissues itself [38]. Exposure to MCP pesticide had no effect on the *d1* mRNA expression in the brain, but stimulated the transcription of *d1* gene in the kidney of female goldfish. The functional roles of D1 might indicate why the patterns of *d1* mRNA regulation varied among tissues after exposure to MCP. Recent studies have suggested that the major role of D1 might be to clear rT_3_ and sulfated iodothyronines from circulation. Indeed, it functions as a scavenger enzyme to remove inactive iodothyronines and recycle iodine within the organisms [39]–[41]. Thus, 0.10 mg/L MCP pesticide might enhance the metabolism of THs by up-regulating the expression of *d1* mRNA in the kidney. Further, the specific patterns of deiodinase-mediated gene regulation show tissue-specific variations, probably in accordance with the distinct thyroid status and tissue-specific requirements for available THs [42]. In the brain, the transcriptions of *d2* and *d3* gene were stimulated after treatment with 0.01 and 0.10 mg/L MCP but inhibited by 1.00 mg/L MCP pesticide, whereas, in the kidney, the transcriptions of *d2* and *d3* gene were stimulated after treatment with 0.10 mg/L MCP but inhibited by treatment with 0.01 and 1.00 mg/L MCP pesticide. Such expression profiles of *d2* and *d3* changing in a parallel way indicates that intracellular T_3_ levels are tightly controlled [43].

THs are also regulated by TSH and TRH/CRH, but can interfere with the synthesis and release of TSH and TRH/CRH *via* feedback mechanisms. This regulatory system plays an important role in maintaining the homeostatic balance along the HPT axis [44], [45]. In the present study, the *tshβ* mRNA levels were up-regulated and showed a negative correlation with plasma TT_3_ levels. The T_3_ feedback mechanism is known to control TSH expression in certain teleosts, and Sohn et al. [46] provided evidence that T_3_ acted directly on the pituitary and inhibited *tshβ* gene expression in goldfish, probably *via* the negative T_3_-responsive elements in the *tshβ* gene. Hence, the decreased plasma TT_3_ levels would feedback to stimulate the transcription of *tshβ*, and similar negative feedback has also been observed in males [9]. TSH is a glycoprotein secreted by the pituitary thyrotrope cells. In our previous study, Bing [47] reported direct damage of the MCP pesticide to the structure of thyrotrope cells in adenohypophysis. The partly dissolved nuclear membrane, prominent dilated rough endoplasmic reticulum, and slightly dissolved mitochondrial cristae indicated a reduction on the hormone secretion yield of thyrotrope cells. Accordingly, in the current study, although pituitary *tshβ* mRNA levels were elevated, the reduced plasma TSH levels in the 0.10 and 1.00 mg/L MCP treatments were probably resulted from the diminished TSH protein synthesis and secretion of thyrotrope cells. In goldfish, both TRH and CRH might be involved in the regulation of the pituitary-thyroid axis, and TRH might act as a multifunctional hypophysiotropic factor. For instance, TRH is a potent stimulator of alpha melanocyte-stimulating hormone release from the pars intermedia cells in various species of teleost fish, including goldfish [48], and is also involved in the regulation of feeding and locomotor behaviors [49]. Therefore, the up-regulated *trh* mRNA expression levels caused by the MCP pesticide would not be the only response of the HPT axis. Instead, CRH might act as a TSH-releasing factor in lower vertebrates [14], and might also be mediated and/or intensified by a concomitant increase in corticosteroids [50]. It is also worth noting that CRH plays important roles in mediating the hypothalamic-pituitary-interrenal axis (HPI axis) to produce cortisol in response to stress, and that cortisol could also tightly control the release of CRH *via* a negative feedback loop [51], [52]. The reductions in plasma cortisol levels in the 0.10 mg/L group would be the result of MCP interference with cortisol synthesis and metabolism at some level. A recent study in our lab revealed that 0.10 mg/L MCP pesticide decreased interrenal synthesis of cortisol and further promoted the metabolism of cortisol, resulting in lower overall cortisol levels and a diminished stress capacity in female zebrafish (*Danio rerio*) [53]. In goldfish, whether CRH acts on thyroid activity either directly or *via* adrenal steroids has yet to be determined; the decreased plasma TT_3_ and cortisol levels would also signal the up-regulation of *crh* gene expression, as observed in the 0.10 mg/L group. On the other hand, in the 1.00 mg/L MCP pesticide group, both plasma cortisol levels and hypothalamic *crh* mRNA levels were decreased, indicating that the high concentration of MCP pesticide disrupted the feedback mechanisms along the HPI axis. Some investigators have suggested that hyperactivity of the cortisol-producing cells associated with severe stress might lead to an inter-renal exhaustion with very low levels of cortisol [54], [55].

Our results showed that the MCP pesticide disturbed the THs homeostasis and interfered with the transport and conversion of THs, synthesis and secretion of pituitary TSHs, and regulation of hypothalamic TRH/CRH in female goldfish. Similar to the findings in males, MCP pesticide decreased plasma TT_3_ and FT_3_ levels with relatively stable plasma TT_4_ levels, and profiles of the relative abundance of deiodinase transcripts were observed in the liver, brain, and kidneys in female goldfish; however, 0.01 mg/L MCP pesticide merely enhanced the plasma FT_4_ levels in females, and gender differences existed in the hepatic deiodinase transcripts at the highest dose. Although the reproductive and thyroidal endocrine systems have been reported to be related to the neuroendocrine control of many complex functions, including growth, metabolism, and reproduction in teleosts [1], [2], [5], and many chemicals, including polychlorinated biphenyl and perfluorooctanoic acid, could simultaneously cause a variety of effects along both endocrine systems [56]–[59], further studies unraveling the interaction between the MCP-induced reproductive axis disruption and thyroidal axis disruption are necessary to explain gender differences observed in the thyroid system response to MCP exposure in goldfish.
